# 3D MRI Analysis of the Lower Legs of Treated Idiopathic Congenital Talipes Equinovarus (Clubfoot)

**DOI:** 10.1371/journal.pone.0054100

**Published:** 2013-01-30

**Authors:** Suzanne L. Duce, Mariella D’Alessandro, Yimeng Du, Baljit Jagpal, Fiona J. Gilbert, Lena Crichton, Simon Barker, J. Martin Collinson, Zosia Miedzybrodzka

**Affiliations:** 1 Division of Biological Chemistry and Drug Discovery, College of Life Sciences, University of Dundee, Dundee, United Kingdom; 2 Clinical Genetics Centre, Ashgrove House, Foresterhill, Aberdeen, United Kingdom; 3 Fulton Building, Division of Mechanical Engineering and Mechatronics, University of Dundee, Nethergate, Dundee, United Kingdom; 4 Aberdeen Biomedical Imaging Centre, University of Aberdeen, Lilian Sutton Building, Foresterhill, Aberdeen, United Kingdom; 5 Aberdeen Maternity Hospital, Cornhill Road, Aberdeen, United Kingdom; 6 Department of Paediatric Orthopaedic Surgery, Royal Aberdeen Children’s Hospital, Foresterhill, Aberdeen, United Kingdom; 7 University of Aberdeen, School of Medical Sciences, Foresterhill, Aberdeen, United Kingdom; 8 Department of Medical Genetics, Polwarth Building, Foresterhill, Aberdeen, United Kingdom; University of California San Francisco, United States of America

## Abstract

**Background:**

Idiopathic congenital talipes equinovarus (CTEV) is the commonest form of clubfoot. Its exact cause is unknown, although it is related to limb development. The aim of this study was to quantify the anatomy of the muscle, subcutaneous fat, tibia, fibula and arteries in the lower legs of teenagers and young adults with CTEV using 3D magnetic resonance imaging (MRI), and thus to investigate the anatomical differences between CTEV participants and controls.

**Methodology/Principal Findings:**

The lower legs of six CTEV (2 bilateral, 4 unilateral) and five control young adults (age 12–28) were imaged using a 3T MRI Philips scanner. 5 of the CTEV participants had undergone soft-tissue and capsular release surgery. 3D T1-weighted and 3D magnetic resonance angiography (MRA) images were acquired. Segmentation software was used for volumetric, anatomical and image analysis. Kolmogorov-Smirnov tests were performed. The volumes of the lower affected leg, muscle, tibia and fibula in unilateral CTEV participants were consistently smaller compared to their contralateral unaffected leg, this was most pronounced in muscle. The proportion of muscle in affected CTEV legs was significantly reduced compared with control and unaffected CTEV legs, whilst proportion of muscular fat increased. No spatial abnormalities in the location or branching of arteries were detected, but hypoplastic anomalies were observed.

**Conclusions/Significance:**

Combining 3D MRI and MRA is effective for quantitatively characterizing CTEV anatomy. Reduction in leg muscle volume appears to be a sensitive marker. Since 5/6 CTEV cases had soft-tissue surgery, further work is required to confirm that the treatment did not affect the MRI features observed. We propose that the proportion of muscle and intra-muscular fat within the lower leg could provide a valuable addition to current clinical CTEV classification. These measures could be useful for clinical care and guiding treatment pathways, as well as treatment research and clinical audit.

## Introduction

Congenital talipes equinovarus (CTEV), also known as ‘clubfoot’, is a relatively common developmental disorder affecting one or both feet, with an incidence of 0.3–7 per 1000 live births [1]. It is characterised by inversion, plantar flexion and adduction of the foot [2]. Treatment typically involves foot manipulation, serial casting, bracing and occasionally Achilles tenotomise and surgery, in the recent past surgical approaches were more common. CTEV is termed idiopathic in the absence of other clinical features. Its aetiology is unknown but various genetic and environmental factors have been suggested [1], including impairment of myogenesis, angiogenesis, chondrogenesis, neurogenesis and ontogenesis; but the precise mechanisms remain unclear. A failure to complete normal embryonic foot rotation, historically known as the ‘arrest of normal development’ hypothesis [3], was observed in an embryological and magnetic resonance imaging (MRI) study of a naturally occurring mutant mouse model of clubfoot [4].

Currently the assessment of CTEV is largely subjective, relying upon observations of clinical features and general measurements [5]–[8]. MRI has been used to study CTEV, predominantly in research to visualise the bones in the feet [9]–[17]. With the suggestion that myogenesis or angiogenesis could be responsible for CTEV, we were interested in using MRI to investigate the effects CTEV have on the lower leg as this may provide clues into CTEV aetiology. Recently two MRI studies of the lower leg have been published [18], [19]. Ippolito *et al* acquired axial images and reported a marked hypoplasia of the muscular tissue and an increase in adipose tissue in the affected legs of unilateral CTEV participants, including neonates [18]. Merrill *et al* conducted three-dimensional magnetic resonance angiography (MRA) on 11 CTEV participants and reported that 40% of the isolated unilateral clubfoot participants had arterial anomalies in their clubfoot lower legs [19]. Their analysis of axial and coronal 2D images of unilateral CTEV lower legs indicated a decrease in muscle, an increase in subcutaneous fat (9/11 cases), and a slight reduction in the length of tibia and fibula of affected CTEV limbs.

In this paper we present a quantitative 3D MRI and MRA, long term, follow up study of the lower leg anatomy of young people with unilateral and bilateral CTEV. 3D digitally segmented anatomical representations were produced from knee to ankle of the muscles, subcutaneous adipose tissue, tibias, fibulas and arteries. 3D MRA was used to investigate potential anomalies in the spatial position, branching and hypoplasia of the arteries in the lower legs. Data from affected CTEV legs were compared with those from unaffected legs of unilateral CTEV participants and with control cohort legs, and the anatomical differences between groups were quantified. The objectives of this study were to i) quantitatively phenotype CTEV anatomy in treated cases approaching skeletal maturity, ii) build 3D anatomical representations, iii) investigate whether these metrics could be use to provide insight into CTEV aetiology and iv) identify metrics that might be used in clinical classification of CTEV to improve either clinical monitoring or prognosis.

## Methods

### Objectives

3D magnetic resonance imaging (MRI) and angiography (MRA) were used to quantify the anatomy of the lower legs of young people treated for idiopathic congenital talipes equinovarus (clubfoot), differences between CTEV participants and controls were investigated.

### Participants

The lower legs of eleven young people (5 controls and 6 CTEV) aged 12–28 years were imaged in this study. The ‘lower leg’ was defined as the region between the knee and the ankle. The CTEV cohort comprised six participants with non-syndromic, idiopathic bilateral (B) or unilateral (U) CTEV (3 males: B1; B2; U4, and 3 females: U1; U2; U3) who had completed treatment. Laterality was respectively right (U1), left (U2, U3, U4), and bilateral (B1, B2). For the unilateral CTEV participants, the legs that showed no clubfoot phenotype were referred to as ‘unaffected’ (Unaff), while the legs with CTEV were referred to as ‘affected’ (Aff). The CTEV cohort ages ranges from 12 to 28 years old, with an average age of 17.8 years. Clinical details and treatment histories are summarised in Table 1 and Table S1. The mild/moderate/severe CTEV grading system described by Harrold and Walker was used [8]. The CTEV participants were assessed by an experienced CTEV specialist orthopaedic surgeon (SB). Case B2 had mild disease and was treated by casting alone, the remaining five cases were considered to have severe disease and they all received surgical treatment. The time elapsed between treatment and the MR scan ranged from 6 years up to 24 years. U1, U2, U3, U4 and B1 were treated with soft tissue and capsular release surgery; U1 and U2 on a single occasion, U3 and U4 had repeated surgery, and B1 had soft tissue and capsular releases followed by bilateral osteotomies. B1, U3 and U4 have continuing impairment of function, with limited ability to run and on-going pain. Five volunteers who had no known limb pathology acted as controls (C) (1 male: C3, plus 4 females: C1; C2; C4; C5). Their ages ranged from 14 to 21 years old (21, 16, 14, 16, and 16 respectively), with an average age of 16.6 years.

**Table 1:** Clinical details of CTEV participants.

ID	Gender	Age at scan	Laterality	Grade	Number of treatments and current status
B1	M	13	Bi	severe	Neonatal strapping and casting. Dennis Browne Boots (DBB). 3× surgery to each side with osteotomy. Remains symptomatic, significant pain with activities of daily living (ADL). Very limited ability to run.
B2	M	13	Bi	mild	Neonatal strapping and casting, DBB. Functioning well. No ADL issues. Able to walk/run.
U1	F	28	Right	severe	Neonatal strapping. Surgery x 1. Functioning well. No ADL issues. Able to walk/run.
U2	F	24	Left	severe	Neonatal strapping, 1× surgery. DBB. Symptoms controlled by insole. No ADL issues.
U3	F	17	Left	severe	Neonatal strapping. DBB. 4× surgery. Functioning well. ADLs no issues. Able to walk, running limited, in pain.
U4	M	12	Left	severe	Neonatal casting. DBB. 2× surgery. Remains symptomatic. Significant pain with ADL. Very limited ability to run.

### Ethics

The study was approved by the NHS North of Scotland Research Ethics committee. The participants were recruited from the Department of Orthopaedic Surgery, NHS Grampian. Informed written consent to participate in this study was obtained from all volunteers and their parents when necessary.

### Magnetic Resonance Imaging

MRI data were acquired on a 3T Philips Achieve scanner (Achieve, Philips Medical Systems, Best, Netherlands) with a torso coil (width 58 cm, length 53 cm). Subjects lay supine and both legs were imaged simultaneously from knee to ankle. Three dimensional T1-weighted Fast Field Echo (FFE) images were acquired with 11 ms repetition time (TR) and 2.3 ms echo time (TE). Typical dataset size was 528 by 528 by 125 and the voxel size was 1 mm by 1 mm by 1 mm. 3D cardiac triggered non-contrast enhanced magnetic resonance angiography (MRA) experiments were performed using the TRANCE pulse sequence [20]. TRANCE method acquired turbo spin echo images during the systole and diastole phase of the cardiac cycle. The systolic and diastolic image datasets were subtracted to produce 3D images showing bright arteries on dark background. The repetition time was 1000 ms, echo time was 65 ms, typical data size were 512 by 512 by 45, and they had similar field of view as the T1-weighted experiments.

### Data Analysis

The Fourier transformed MRI were visualised, and anatomical regions of interest were digitally segmented using the Amira software (Visage Imaging GmbH, 12163 Berlin, Germany). 2D images could be viewed from any orientation within the 3D datasets. 3D surface representations were produced by surface rendering the 3D finite element meshes generated from segmenting the regions of interest. The entire 3D T1-weighted MRI data of lower legs were used to digitally segment the tibias and fibulas. Whilst the regions assigned as the lower leg were segmented from the axial plane just above the proximal tibial plateau to the axial plane just below the distal tibial plafond. After which, the muscles and subcutaneous adipose tissues were segmented from the axial plane just above the proximal tibial physis to the axial plane near to the proximal region of the Achilles tendon above the distal tibial physis. Other anatomical features such as the nerve bundles and tendons were not segmented. The segmentation was semi-automated using both automatic signal intensity thresholding and manual painting methods. The volumes of the lower leg (V_Leg_), the lower leg muscles (V_Musc_), subcutaneous fat (V_Fat_), tibia (V_Tibia_) and fibula (V_Fibula_) were determined from the 3D segmentation. The MRA experiments produced 3D image datasets where high intensity signal arises from arterial blood. Automated signal intensity thresholding methods were used to digitally segment the popliteal, anterior tibial, posterior tibial, and peroneal arteries.

To investigate the soft tissue composition of the lower legs, the percentages of muscle (Muscle%) and subcutaneous fat (Fat%) were calculated using the equation:
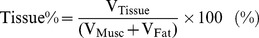
(1)where V_Tissue_ is either the volume of fat (V_Fat_) or muscle (V_Musc_). In this study, the proportion of high intensity pixels in the images of lower leg muscles (TA_Musc_) originating predominantly from intra- and inter-muscular adipose tissue was estimated using the formula:
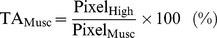
(2)where PixelMusc is the total number of pixels in the lower leg muscle, and PixelHigh is the number of pixels where signal intensity was over 1.5 times higher than neighbouring muscle pixel intensity.

The image segmentation protocols were not fully automated which can result in operator error. An inter-operator reproducibility study was completed using three trained operators and the results present in . It demonstrated that the fractional error of mean (FEM) of the segmentation protocol were below 4% for the muscle, subcutaneous fat and tibia measurements, and under 9% for the fibula measurements. These values were considered acceptable. The largest errors were associated with measuring fibula volumes. Both shape and image contrast are important factors regarding reproducibility, and the fibula’s large surface area was a major source error.

### Statistical Methods

Mean and standard deviations (SD) were calculated and quoted as mean ± SD, interquartile ranges were calculated and shown in box plots. The Kolmogorov-Smirnov (KS) test was used to identify whether the test data were significantly different from control data. The KS test calculates maximal distance (D) between cumulative distributions, an in-house software package was utilised (Data Analysis Group, University of Dundee). This statistical method is particularly suitable when datasets are small or unequally dispersed. The null hypothesis asked whether the test and control data were drawn from the same population, and a *P* value of less than 0.05 was considered statistically significant. Inevitably, the quality of the MRI experimental data was degraded by leg movements during the experiment, radio-frequency coil and magnetic field inhomogeneities, partial volume effects either at tissue boundaries or from microscopic anatomical features, and water and lipid chemical shift artefacts. Despite this, the quality of the anatomical information in these 3T images was very good. In T1-weighted images, the signal-to-noise of subcutaneous fat and muscle was of the order of 180∶1 and 57∶1 respectively; whilst the contrast between muscular tissue and adjacent subcutaneous adipose tissue and adjacent cortical bone was of the order 0.52 and 0.75 respectively [21].

## Results

### 1. Anatomical T1-weighted MRI

**Figure 1:**
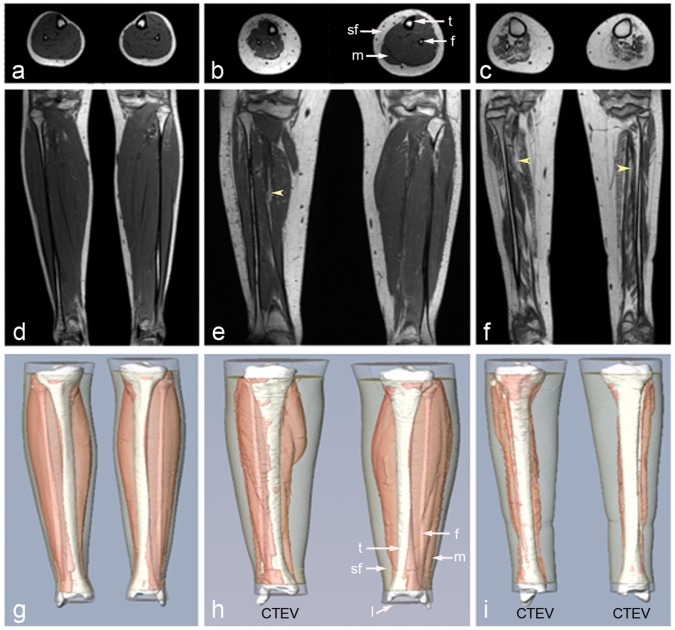
3D T1-weighted MRI images of control, unilateral and bilateral CTEV legs. (a,d,g) Control male (C3); (b,e,h) Unilateral CTEV female (U1); (c,f,i) Bilateral CTEV male (B1). (a–c) Axial distal images at widest part of the lower leg; (d–f) Anterior coronal images through the fibula; (g–i) Anterior coronal views of 3D surface reconstructions of lower leg (transparent white), subcutaneous fat (transparent cream), muscular tissue (pink), tibia and fibula (white). t = tibia, f = fibula, m = muscle, sf = subcutaneous fat, yellow arrows points to muscular fat. T_R_/T_E_ = 11/2.3 ms; matrix size = 528 by 528 by 125; spatial resolution of voxel = 1 mm by 1 mm by 1 mm.

Representative coronal and axial T1-weighted images of control, unilateral and bilateral CTEV volunteers are displayed in Figure 1a–f. The lower legs, their muscles, subcutaneous fat, tibias, and fibulas were segmented, and typical 3D surface representations of the anatomical regions of interest are shown in Figure 1g–i. The volumetric results from the lower leg (V_Leg_) were reported in Table 2. The subcutaneous fat (V_Fat_) and muscle (V_Musc_) volumes were recorded in Table 3. The tibias (V_Tibia_) and fibulas (V_Fibula_) values were tabulated in Table 4. The resolution of the 3D MR scans limited the detailed anatomical characterisation to muscle compartment rather than allowing delineation of individual muscle groups.

**Table 2:** Lower leg volumes of control and CTEV participants, with ratio calculations.

	V_Leg_ (cm^3^)	V_Leg_ (cm^3^)	V_leg_
Control	Right	Left	Right:Left
C1	2511.1	2499.1	1.0
C2	2221.4	2293.1	0.97
C3	2382.7	2268.8	1.05
C4	2922.9	2947.3	0.99
C5	1993.2	2000.2	1.0
**Bi-CTEV**	**Right**	**Left**	**Right:Left**
B1	2278.3	2244.4	1.02
B2	2595.4	2622.0	0.99
**Uni-CTEV**	**Aff**	**Unaff**	**Aff:Unaff**
U1	3011.8	3275.2	0.92
U2	2724.4	3219.5	0.85
U3	1240.5	1717.0	0.72
U4	1291.7	1551.7	0.83

V_Leg_ = lower leg volume, Aff = affected, Unaff = unaffected.

**Table 3:** Muscle and subcutaneous fat volumes in the lower legs of control and CTEV participants, with soft tissue and ratio calculations.

	V_Musc_ (cm^3^)		V_Fat_ (cm^3^)		Muscle%		Fat%		Muscle	Fat
Control	Right	Left	Right	Left	Right	Left	Right	Left	Right:Left	Right:Left
C1	1262.4	1233.8	765.5	736.3	62.3	62.6	37.7	37.4	1.02	1.04
C2	968.3	982.7	748.3	802.5	56.4	55.0	43.6	45.0	0.99	0.93
C3	1271.0	1192.7	576.0	589.9	68.8	66.9	31.2	33.1	1.07	0.98
C4	1203.8	1181.2	1157.2	1186.9	51	49.9	49.0	50.1	1.02	0.98
C5	1010.4	1029.4	607.8	564.5	62.4	64.6	37.6	35.4	0.98	1.08
**BiCTEV**	**Right**	**Left**	**Right**	**Left**	**Right**	**Left**	**Right**	**Left**	**Right:Left**	**Right:Left**
B1	343.0	236.6	1344.7	1389.8	20.3	14.5	79.7	85.5	1.45	0.97
B2	1052.6	1080.8	1000.5	1008.9	51.3	51.7	48.7	48.3	0.97	0.99
**UniCTEV**	**Aff**	**Unaff**	**Aff**	**Unaff**	**Aff**	**Unaff**	**Aff**	**Unaff**	**Aff:Unaff**	**Aff:Unaff**
U1	882.9	1358.8	1511.3	1309.6	36.9	50.9	63.1	49.1	0.65	1.15
U2	940.8	1488.2	1232.0	1183.6	43.3	55.7	56.7	44.3	0.63	1.04
U3	410.7	854.7	488.7	486.5	45.7	63.7	54.3	36.3	0.48	1.0
U4	440.4	712.5	392.7	387.0	52.9	64.8	47.1	35.2	0.62	1.01

V_Fat_ = subcutaneous fat volume, V_Musc_ = muscle volume, Aff = affected, Unaff = unaffected.

**Table 4:** Tibia and fibula volumes of control and CTEV participants, with ratio calculations.

	V_Tibia_(cm^3^)		V_Tibia_	V_Fibula_ (cm^3^)		V_Fibula_
Control	Right	Left	Right:Left	Right	Left	Right:Left
C1	249.0	251.8	0.99	46.2	46.6	0.99
C2	241.1	241.9	1.0	42.4	40.4	1.05
C3	284.1	271.6	1.05	41.9	41.7	1.01
C4	270.4	269.0	1.01	51.1	51.2	1.0
C5	203.3	198.4	1.02	34.7	32.4	1.07
**Bi-CTEV**	**Right**	**Left**	**Right:Left**	**Right**	**Left**	**Right:Left**
B1	288.6	320.9	0.9	57.9	52.0	1.11
B2	287.1	289.4	0.99	41.3	42.6	0.97
**Uni-CTEV**	**Aff**	**Unaff**	**Aff:Unaff**	**Aff**	**Unaff**	**Aff:Unaff**
U1	216.4	247.0	0.88	28.18	29.2	0.96
U2	261.1	273.1	0.96	41.1	47.2	0.87
U3	159.4	187.0	0.85	16.4	20.6	0.8
U4	250.5	256.7	0.98	33.4	36.7	0.91

V_Tibia_ = tibia volume, V_Fibula_ = fibula volume, Aff = affected, Unaff = unaffected.

#### 1.1. Contralateral leg study of control and unilateral CTEV cohorts

**Figure 2:**
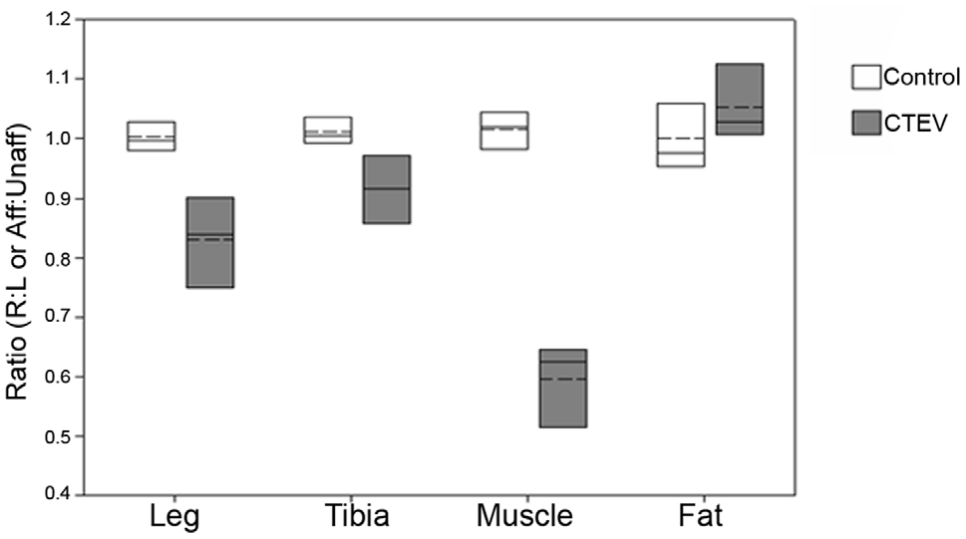
Right-to-left and affected-to-unaffected ratios of lower legs, tibias, muscles or subcutaneous fat volumes. Ratio of right-to-left (R:L) volumes for controls (n = 5) (white). Affected-to-unaffected (Aff:Unaff) volumes for the unilateral CTEV group (n = 4) (grey). The mean (dashed line) and median (solid line) values are shown within boxes representing 25% and 75% limits.

For the unilateral CTEV cohort (n = 4), the ratio of the affected/unaffected volumes (Aff:Unaff) of lower leg, subcutaneous fat, muscle, tibia and fibula were calculated. In the control group, the ratios of the right/left volumes (R:L) were calculated (n = 5). The results were illustrated in Figure 2.

The average ratio of right/left lower leg volumes in the control group was 1.00±0.03, showing no overall left-right bias (Table 2). Leg asymmetry was observed in individuals within the controls; for example C2’s lower right leg was 3.1% smaller than her left, and C3’s left leg was 4.8% smaller than his right. Interestingly, C2 is left handed and C3 is right handed. For the unilateral CTEV participants, the average ratio of affected/unaffected lower leg volumes was 0.83±0.081; the affected lower legs were from 8.7 to 38.4% smaller than the contralateral unaffected legs. The differences between the control and unilateral CTEV results were statistically significant (KS test: *P* = 0.0069, D = 1).

The average ratio of right/left muscle volumes in the control group was 1.01±0.034 (Table 3), with differences in contralateral muscle volumes ranging from 1.5 to 6.2%. The average ratio of right/left subcutaneous fat volumes in the control group was 1.00±0.057. For the unilateral CTEV participants, the volumes of the muscles in the affected legs were significantly smaller than those of the contralateral unaffected legs. The average muscle volume ratio of the affected/unaffected lower leg was 0.6±0.078, with the affected muscles being from 35 to 52% smaller than the contralateral unaffected leg. The average subcutaneous fat volume ratio of the affected/unaffected lower leg was 1.05±0.069. The differences between the control and unilateral CTEV muscle results were statistically significant (*P* = 0.0069, D = 1), but differences were not significant for subcutaneous fat (*P* = 0.259, D = 0.6) probably because it was underpowered.

The average ratio of the right/left tibia and fibula volumes in the control legs were 1.01±0.023 and 1.02±0.034 respectively (Table 4). On average there was a slight right bias, with the tibia volumes differing on average by about 1%. In contrast in the unilateral CTEV group, the average ratio of affected/unaffected tibia and fibula volumes were 0.92±0.06 and 0.89±0.068 respectively. The volumes of the affected CTEV tibias were on average 8% smaller than their contralateral unaffected tibias. The differences in the tibia (*P* = 0.0069 and D = 1) and fibula (*P* = 0.0069, D = 1) volumes were statistically significant.

#### 1.2. Comparison between the affected CTEV, unaffected CTEV and control legs

**Figure 3:**
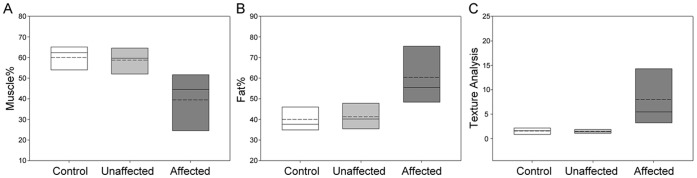
Comparison between anatomy of control, unaffected and affected CTEV legs. (A) Percentages of muscle and (B) subcutaneous fat in soft tissue of lower legs, and (C) image analysis (TA_musc_) of muscular fat in lower leg muscles images. The control legs (n = 10) (white), unaffected CTEV legs (n = 4) (pale grey), and affected CTEV legs (n = 9) (dark grey). The mean (dashed line) and median (solid line) values are shown within boxes representing 25% and 75% limits.

The percentages of muscle (Muscle%) and subcutaneous fat (Fat%) in the soft tissues of the controls and CTEV lower legs were calculated (Equation 1), and recorded in Table 3. The averaged percentages of muscle in the control lower legs (n = 10) and CTEV unaffected lower legs (n = 4) were similar at 60±6.5% and 58.8±6.6% respectively. In contrast the averaged percentage of lower leg muscle for each participant (n = 6) was significantly lower (41.3.6±13.1%) in the CTEV affected legs compared to the controls, as illustrated in Figure 3A and B. The differences were statistically significant (*P* = 0.0066 and D = 0.8).

Close examination of the T1-weighted images suggested that the CTEV lower leg muscles contained a higher proportion of regions with high intensity signal (producing a bright ‘speckled’ appearance) compared to control or unaffected CTEV images (see yellow arrows in Figure 2e and f). This signal originates predominantly from adipose tissue. The proportions of high signal intensity pixels in the muscular tissues (TA_Musc_) were quantified (Equation 2), recorded in Table 5 and graphically displayed in Figure 3C. The average TA_Musc_ of the control legs (n = 10) and unaffected CTEV legs (n = 4) were 1.53±0.65% and 1.46±0.44% respectively. The average TA_Musc_ of bilateral CTEV affected legs muscle were used in calculations. Whilst the proportion of muscular fat (TA_Musc_) in the lower legs of the control and unaffected unilateral legs were quite similar, in contrast the average TA_Musc_ for affected legs in each CTEV participants (n = 6) was markedly higher at 7.2±5.64%. The differences between the control and affected CTEV legs were statistically significant (*P* is 0.00026, D = 1).

**Table 5:** Proportion of high intensity pixels (TA_Musc_) in the images of lower leg muscles of control and CTEV participants, with ratio calculations.

	TA_Musc_	TA_Musc_	TA_Musc_
Control	Right	Left	Right:Left
C1	0.63	1.09	0.57
C2	2.2	2.36	0.93
C3	0.9	0.73	1.23
C4	1.54	1.68	0.91
C5	2.12	2.03	1.05
**Bi-CTEV**	**Right**	**Left**	**Right:Left**
B1	16.38	19.71	0.83
B2	2.43	3.15	0.77
**Uni-CTEV**	**Aff**	**Unaff**	**Aff:Unaff**
U1	4.8	1.77	2.72
U2	3.38	1.1	3.08
U3	6.21	1.08	5.73
U4	7.88	1.91	4.12

TA_musc_ = image analysis, Aff = affected, Unaff = unaffected.

### 2. 3D Magnetic Resonance Angiography (MRA)

**Figure 4:**
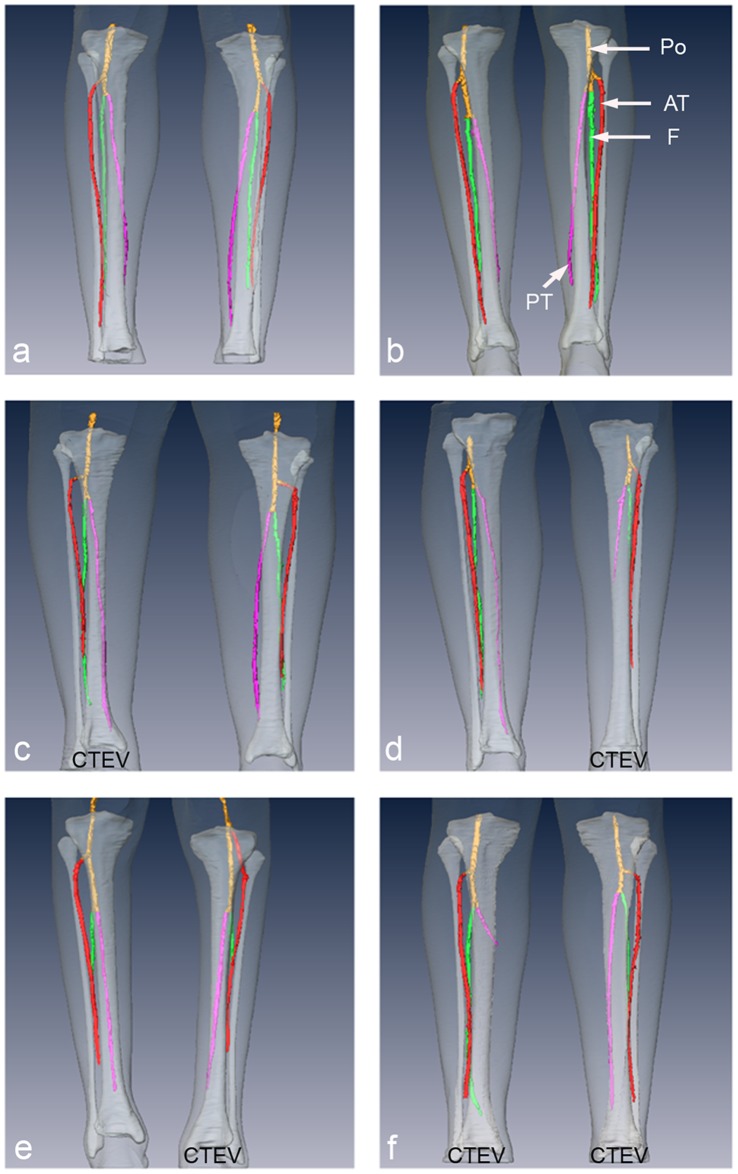
Anterior coronal 3D MRA reconstructions of arteries in legs of CTEV and control teenagers and young adults. The popliteral artery ‘Po’ (yellow), anterior tibia artery ‘AT’ (red), posterior tibia artery ‘PT’ (pink), and fibula artery ‘F’ (green) are overlaid onto 3D surface reconstructions of lower leg (transparent white) and tibia and fibula (white) from T1-weighted MRI. (a) C3, (b) C5, (c) U1, (d) U2, (e) U4, (f) B2.

3D non-contrast enhanced MRA images of control (n = 2), unilateral CTEV (n = 3) and bilateral CTEV (n = 1) participants were acquired. Anterior MRA images of the popliteal, anterior tibial, posterior tibial, and peroneal arteries were displayed in Figure 4. The posterior views are shown in Figure S1. To provide anatomical landmarks, the arterial images were viewed alongside the 3D anatomical representations of the lower leg, tibia and fibula from the T1-weighted MRI study. Anomalies in the 3D spatial position, branching and hypoplasia of the arteries in the lower legs were investigated. The arteries were viewed from all angles, and the arteries in affected CTEV lower legs were compared with contralateral and control legs. No spatial abnormalities in location or branching of arteries in CTEV legs were detected. However, there were anomalies involving hypoplasia associated with underdevelopment of arteries. The results were tabulated (Table 6), 3 out of 5 of the affected CTEV legs showed hypoplasia. In comparison, 1 out of 7 unaffected legs showed hypoplasia; this leg was the unaffected leg of unilateral CTEV participant (U4).

**Table 6:** Results from MRA analysis of the anomalies in the main lower leg arteries of control and CTEV participants.

	Hypoplasia Arterial Anomalies	
Control	Right	Left
C3	None	None
C5	None	None
**CTEV**	**Affected**	**Unaffected**
U1	None	None
U2	Posterior tibialand Fibular arteries	None
U4	Fibular artery	Fibular artery
B2-right	Posterior tibial artery	–
B2-left	None	–

## Discussion

This paper reports the first three-dimensional anatomical segmentation of the entire lower legs of young people with CTEV using MRI. Previous MRI studies acquired multi-slice datasets and analysed individual slices through the leg [18], [19]. In this study, the volume of the lower legs, muscles, subcutaneous fat, tibias and fibulas were determined from the segmentation of continuous 3D MRI datasets encompassing the lower leg from knee to ankle. The 3D volumetric anatomical analysis demonstrated that the volumes of the affected lower legs of the unilateral CTEV participants, their muscles, tibias and fibulas were consistently smaller than their contralateral unaffected limb. The muscle volumes were much more sensitive to CTEV than the total lower leg volume. Muscle hypoplasia and atrophy are recognised features of CTEV, although their effect on leg circumferences is often masked by an increase in subcutaneous fat. An example of this is bilateral CTEV participant B1, the total volume of the lower right leg was only 1.5% larger than the left leg, suggesting that the legs are quite similar. However, his right lower leg muscle volume was 45% larger than his left.

In this study the volumes of the whole tibia and fibula were determined. On average the volume of the tibia in the affected leg of unilateral CTEV participants was 8.5% smaller than that of the contralateral unaffected leg. Differences in the length and cross-sectional, axial area of tibias and fibulas in contralateral legs of unilateral clubfoot patients have been reported previously [18], [19], [22]–[25]; whilst this is seldom observed in the general population [26]. The differences have been attributed to surgical treatment [23] and to decrease in foot height [25], although it is more likely they are due to asymmetric limb development.

In Table 1 and Table S1 we summarise the clinical information available for each participant. Formal pre-treatment grading was done using the previously widely adopted, pragmatic, qualitative clinical scale of mild/moderate/severe as described by Harrold and Walker [8]. Our currently preferred Pirani score had not been described at the time this cohort were first clinically assessed [7]. The participants had ‘typical’ non-syndromic/idiopathic CTEV; with one (B1) being so severe that bone surgery had been necessary. Four others, with the exception of B2 who was treated by casting alone, had capsular releases and soft-tissue surgery, sometimes on multiple occasions but no muscles were redirected. Whilst we acknowledge that the features described could be the result of the surgical treatment, it is likely that the changes observed in the MRI are the result of the developmental pathogenesis of CTEV. In all the surgical procedures, significantly tendons were not merely divided but elongated with the aim of restoring functional length to the tendon. This in turn helps to normalise the forces expected to act on the respective muscle. Therefore it is anticipated that the forces acting on the muscles will provide a more normal stimulus for growth, especially compared to the case of lower limb development in the absence of treatment. Periods of leg immobilisation during treatment are inevitable and detailed in Table S1. The maximum period of immobilisation for these subjects was 2 years, much shorter than the accepted current treatment with the Ponseti technique. The time period from completion of immobilisation to the MRI scan was from 6 to 24 years. We propose that our findings can primarily be attributed to the developmental pathogenesis of the condition; they are not simply the result of either differences in weight bearing; surgical treatments; muscle immobilisation; or a consequence of iatrogenic removal of muscle tension. A comparative MRI study of young adults treated by Ponseti technique would be interesting, as it would help clarify this issue. Only in recent years has Ponseti become the method of choice for CTEV treatment, and as a result young adults treated by this method alone were not available for recruitment to this study.

In this study, the increase in adipose tissue within the muscle of affected CTEV legs was observed, due to either muscle atrophy or hypoplasia. Other lipids such as those in the myelin sheath surrounding the axons will also contribute to the high intensity signal, and the inevitable partial volume effects will detrimentally affect the result. Ippolito *et al* reported the presence of fat in the interstitial regions of the atrophied muscles [18]. It is interesting that in unilateral CTEV participants, the presence of adipose tissue in the muscle of the affected leg is much higher than in the contralateral unaffected leg. The potential to use such a measure as a prognostic marker of muscle function merits further work.

This is the first reported 3D segmentation of CTEV MRA images, previous MRA clubfoot studies presented maximum intensity projections [19], [27]. Segmentation allowed representation of the main arteries to be visualised alongside those of the muscles, subcutaneous fat, tibias and fibulas in the lower legs; thus producing digital 3D anatomical models of CTEV lower legs. Our 3D MR angiography revealed no spatial abnormalities in the location or branching of the main arteries in CTEV lower legs. Hypoplasia of the arteries was observed in affected CTEV legs at a higher frequency (3/5) compared to the unaffected unilateral legs (1/3) and control legs (0/4). Whilst congenital vascular anomalies have been observed in approximately 8% of healthy individuals [28], [29], abnormalities in the arterial structure have been reported to occur at a much higher frequency in clubfoot patients than in the general population [19], [27], [28], [30]–[32]. A MRA study by Merrill *et al* reported 4 out of 10 isolated unilateral clubfoot patients had arterial anomalies which include arterial hypoplasia [19]. Non-contrast enhanced MRA has the advantage that it does not require the injection of any exogenous contrast agent. It does involve the subtraction of two images to produce a difference image, and this has the disadvantage that any leg movements during imaging can introduce artefacts and degrade image quality. Also it should be noted our group size was quite small.

Often the unaffected legs of unilateral CTEV patients are assumed to be ‘normal’; although the validity of this presumption has not been previously tested. In this paper we investigated this hypothesis by measuring the proportion of muscle in the soft tissues and muscular fat of the leg. The data from the unaffected legs of unilateral CTEV participants were similar to those of normal legs, and quite different to those of affected CTEV legs. However although the anatomy of the unaffected legs of unilateral CTEV participants closely resembled that of normal legs, this does not prove they are completely ‘normal’.

There are a number of ways to extend and improve this 3D MRI CTEV study. The number of participants in the groups could be increased to establish the validity of the hypotheses about causality and to assess the value of these proposed measures in clinical practice. With increased numbers, it would also be feasible to subdivide the groups by gender, age and on the basis of their CTEV severity. Other clinical information such as whether the participants were right or left handed, and the current exercise regime of the individual could also be collated. A complementary study that could clarify if these findings are associated with primary aetiology of CTEV would be to image pre-treated CTEV neonates. This would avoid the limitations associated with a imaging study of young adult limbs that have undergone treatment.

In conclusion, this study has demonstrated that 3D anatomical representations can be produced by segmenting 3D T1-weighted MRI and the 3D MRA datasets from the lower leg of young people with CTEV. This is a very powerful method for visualising and quantifying clubfoot anatomy. The volume of lower leg muscle is particularly affected by CTEV, and the proportion of muscle in the soft tissue of CTEV legs was significantly lower than in controls, whilst the proportion of adipose tissue was higher. The phenotype of the unaffected lower leg of unilateral CTEV participants more closely resembled those of normal legs than those of affected CTEV legs. We propose that the changes we have observed are primarily due to the underlying developmental pathogenesis of CTEV, and are not simply the result of surgery, other treatments or weight bearing. To unequivocally clarify this more research is required, which could be obtained by quantifying the soft tissues in the lower legs of young adults who were treated by Ponseti method and also of neonates with CTEV prior to their treatment.

Several metrics developed in this paper may be useful in the assessment and management of CTEV. This was a small clinical study and thus further research is required to evaluate their general clinical utility, and to refine the techniques. For example it would be advantageous to achieve shorter data acquisition times and fully automated image analysis. This could be a valuable addition to current scoring systems to improve clubfoot management, by informing prognostication, guiding treatment pathways and in clinical treatment audit.

## Supporting Information

Figure S1 **Posterior coronal 3D MRA reconstructions of arteries in legs of CTEV and control young adults.** The popliteral artery ‘Po’ (yellow), anterior tibia artery ‘AT’ (red), posterior tibia artery ‘PT’ (pink), and fibula artery ‘F’ (green) are overlaid onto 3D surface reconstructions of lower leg (transparent white) and tibia and fibula (white) from T1-weighted MRI. (a) C3, (b) C5, (c) U1, (d) U2, (e) U4, (f) B2.
(TIF)

Table S1 **Clinical treatment history of CTEV participants.**
(DOCX)

Table S2 **Reproducibility study of volumetric segmentation protocol.** The volumes (V_Fat_, V_Musc_, V_Tibia_ and V_Fibula_) were determined form control (C3) and bilateral CTEV (B1) lower leg datasets by 3 trained operators. The fractional error of mean (FEM) is shown.
(DOCX)
