# Supplementary figures and images for: 3D MRI Analysis of the Lower Legs of Treated Idiopathic Congenital Talipes Equinovarus (Clubfoot)
Source: PLoS One. 2013 Jan 30;8(1):e54100. doi: 10.1371/journal.pone.0054100 (PMC3559654; doi:10.1371/journal.pone.0054100)

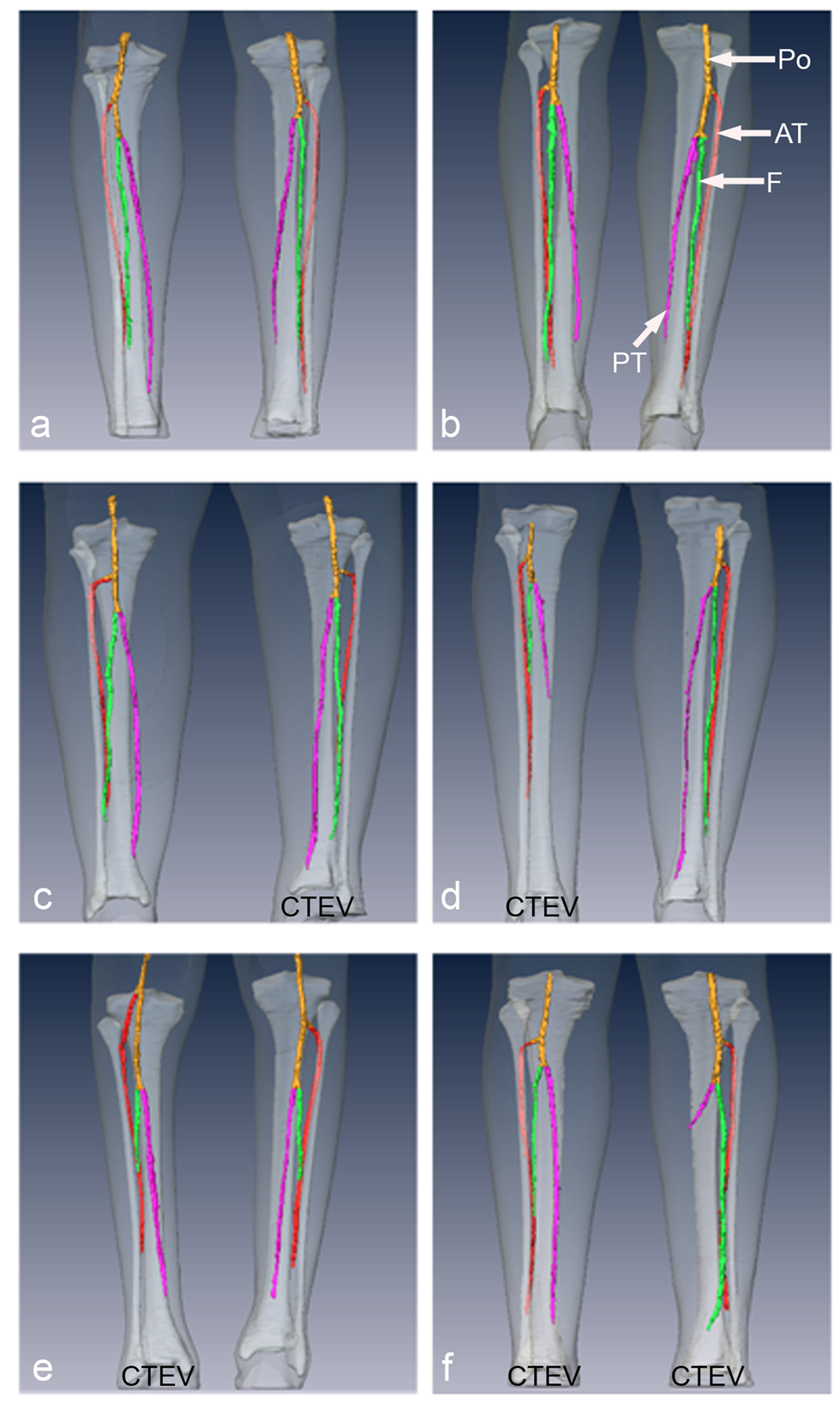

Supplement: Figure S1 — Posterior coronal 3D MRA reconstructions of arteries in legs of CTEV and control young adults. The popliteral artery ‘Po’ (yellow), anterior tibia artery ‘AT’ (red), posterior tibia artery ‘PT’ (pink), and fibula artery ‘F’ (green) are overlaid onto 3D surface reconstructions of lower leg (transparent white) and tibia and fibula (white) from T1-weighted MRI. (a) C3, (b) C5, (c) U1, (d) U2, (e) U4, (f) B2. (TIF) [file pone.0054100.s001.tif]
