# Supplementary material for: 3D MRI Analysis of the Lower Legs of Treated Idiopathic Congenital Talipes Equinovarus (Clubfoot)
Source: PLoS One. 2013 Jan 30;8(1):e54100. doi: 10.1371/journal.pone.0054100 (PMC3559654; doi:10.1371/journal.pone.0054100)
Supplement: Table S1 — Clinical treatment history of CTEV participants. (DOCX) [file pone.0054100.s002.docx]

**Supplementary Table S1**. Clinical treatment history of CTEV participants.

| **ID** | **Treatment details** | **Functional outcome** |
| --- | --- | --- |
| **B1** | *Neonatal* Strapping | Remains symptomatic |
|  | *2 months (mo)* Plaster cast | Significant pain with activities of daily living (ADL) |
|  | *5 mo* Bilateral soft tissue release: lengthening Tendo-Achilles and Tibialis posterior. Turco type Postero-medial release | Very limited ability to run |
|  | *7 mo* Cast change |  |
|  | *13 mo* DBB |  |
|  | *5 yr 10 mo* Dwyer L, Cast change |  |
|  | *6 yr 4 mo* Dwyer R |  |
|  | *6 yr 5 mo* cast change |  |
|  | *8 yr 7 mo* Redo Dwyer opening wedge, R osteotomy |  |
|  | *8 yr 11 mo* Redo L Dwyer |  |
| **B2** | *Neonatal* Strapping | Functioning well |
|  | *1 mo* Serial casting (not Ponseti) | No ADL issues |
|  | *2 mo* DBB | Able to walk/run |
| **U1** | *Neonatal* Strapping*.* Poor compliance with treatment | Functioning well |
|  | *6 yr* Soft tissue release R: ‘full postero-medial release’ (no further detail available) | No ADL issues, able to walk/run |
|  |  |  |
| **U2** | *Neonatal* manipulation and strapping | Symptoms controlled by insole |
|  | *2 mo* Postero-medial release: Tibialis posterior lengthened Tendo Achilles lengthened | No ADL issues |
|  | *3 mo* DBB |  |
|  | *11 mo* Boots and bar fitting well |  |
|  | *12 mo* DBB abandoned by patient |  |
|  | *2 yr 10 mo* Satisfactory |  |
|  | *7 yr* L foot pain – no cause found |  |
|  | *7 yr 10 mo* L foot 1.5 sizes smaller |  |
|  | *8 yr* Heel pain Severe disease bilaterally |  |
|  | *9 yr* Heels settled |  |
|  | *10 yr* Discharged – ‘excellent’ |  |
|  | *10 yr* Discharged – ‘excellent’ |  |
| **U3** | *Neonatal* Strapping | Functioning well |
|  | *4mo* Soft tissue surgery: Tendo-Achilles lengthening, Elongation Tibialis posterior, capsulotomy ankle, sub-talar & talo-navicular joints | ADLs – no issues |
|  | *7 mo* Dennis Browne Boots (DBB) fitted but not tolerated. Long leg cast | Able to walk, running limited, in pain |
|  | *9* *mo* Night splint *10 mo* DBB |  |
|  | *12 mo* Relapsed; Redo Soft tissue release: Tendo achilles elongated, Ankle & sub-talar capsulotomy, Talo-fibular & calcaneo-fibular ligs part divided, Talo-navicular release |  |
|  | *1 yr 3mo* night splints |  |
|  | *1 yr 4 mo* Piedro boots |  |
|  | *6yr, 3mo* Steindler release of plantar fascia |  |
|  | *13 yr* Moulded insole |  |
|  | *13 yr, 9mo* Leg length discrepancy 2.25cm |  |
|  | *14 yr* 8 plates R tibia (for limb lengthening device (LLD)) |  |
|  | *15 yr* 8 plates R femur (for LLD) |  |
|  | *16 yr* Removal R femoral / tibial 8 plates |  |
| **U4** | *Neonatal* Strapping | Remains symptomatic |
|  | *2.5 mo* Plaster cast | significant pain with ADL |
|  | *3.5 mo* Soft tissue release: Tendo Achilles & tibialis post lengthened, ankle & sub-talar joint released, talo-navicular joint released, calcaneo-fibular & interosseous ligament released | Very limited ability to run |
|  | *4 mo* Cast change *5 mo* DBB |  |
|  | *21 mo* Soft tissue release: Ankle & soft tissue capsulotomy |  |
|  | *21.5 mo* change of cast *23 mo* DBB |  |
