# Supplementary material for: 3D MRI Analysis of the Lower Legs of Treated Idiopathic Congenital Talipes Equinovarus (Clubfoot)
Source: PLoS One. 2013 Jan 30;8(1):e54100. doi: 10.1371/journal.pone.0054100 (PMC3559654; doi:10.1371/journal.pone.0054100)
Supplement: Table S2 — Reproducibility study of volumetric segmentation protocol. The volumes (VFat, VMusc, VTibia and VFibula) were determined form control (C3) and bilateral CTEV (B1) lower leg datasets by 3 trained operators. The fractional error of mean (FEM) is shown. (DOCX) [file pone.0054100.s003.docx]

**Supplementary Table S2.** Reproducibility study of volumetric segmentation protocol. The volumes (V_Fat_, V_Musc_, V_Tibia_ and V_Fibula_) were determined form control (C3) and bilateral CTEV (B1) lower leg datasets by 3 trained operators. The fractional error of mean (FEM) is shown.

|  | **FEM_Fat_ (%)** | **FEM_Musc_ (%)** | **FEM_Tibia_ (%)** | **FEM_Fibula_ (%)** |
| --- | --- | --- | --- | --- |
| **C3 (Right)** | 1.21 | 0.65 | 0.96 | 2.28 |
| **C3 (Left)** | 1.68 | 0.59 | 2.92 | 3.82 |
| **B1 (Right)** | 1.29 | 3.8 | 1.03 | 6 |
| **B1 (Left)** | 0.86 | 2.15 | 1.73 | 8.27 |
| **FEM(%) ± sd** | 1.26 ±0.34 | 1.80 ±1.52 | 1.66 ±0.91 | 5.09 ±2.61 |
